# Metabolic and Transcriptomic Changes in the Mouse Brain in Response to Short-Term High-Fat Metabolic Stress

**DOI:** 10.3390/metabo13030407

**Published:** 2023-03-09

**Authors:** Ji-Kwang Kim, Sehoon Hong, Jina Park, Seyun Kim

**Affiliations:** 1 Department of Biological Sciences, Korea Advanced Institute of Science and Technology (KAIST), Daejeon 34141, Republic of Korea; 2 KAIST Institute for the BioCentury, KAIST, Daejeon 34141, Republic of Korea; 3 KAIST Stem Cell Center, KAIST, Daejeon 34141, Republic of Korea

**Keywords:** obesity, brain, transcriptomics, metabolomics, metabolism pathway

## Abstract

The chronic consumption of diets rich in saturated fats leads to obesity and associated metabolic disorders including diabetes and atherosclerosis. Intake of a high-fat diet (HFD) is also recognized to dysregulate neural functions such as cognition, mood, and behavior. However, the effects of short-term high-fat diets on the brain are elusive. Here, we investigated molecular changes in the mouse brain following an acute HFD for 10 days by employing RNA sequencing and metabolomics profiling. Aberrant expressions of 92 genes were detected in the brain tissues of acute HFD-exposed mice. The differentially expressed genes were enriched for various pathways and processes such as superoxide metabolism. In our global metabolomic profiling, a total of 59 metabolites were significantly altered by the acute HFD. Metabolic pathways upregulated from HFD-exposed brain tissues relative to control samples included oxidative stress, oxidized polyunsaturated fatty acids, amino acid metabolism (e.g., branched-chain amino acid catabolism, and lysine metabolism), and the gut microbiome. Acute HFD also elevated levels of N-acetylated amino acids, urea cycle metabolites, and uracil metabolites, further suggesting complex changes in nitrogen metabolism. The observed molecular events in the present study provide a valuable resource that can help us better understand how acute HFD stress impacts brain homeostasis.

## 1. Introduction

Increasing rates of obesity and related metabolic disorders in recent decades have become a serious public health concern [1,2]. Obesity is a chronic disease that is closely linked to major comorbidities such as type two diabetes, hypertension, and certain types of cancer [3]. Although obesity is considered a multifactorial disorder with genetic dispositions and environmental components (e.g., a sedentary lifestyle), it is ultimately caused by an energy imbalance when calorie intake exceeds energy expenditure, which has been largely attributed to the consumption of diets rich in fat and sugars [4]. Particularly, the prolonged consumption of a high-fat diet (HFD), which increases food and caloric intake per meal, has been demonstrated to induce weight gain and increase adiposity in both human and animal models [5,6].

HFDs are characterized by a high content of saturated fatty acids such as palmitate and stearate. The physiological impacts of prolonged HFD exposure and diet-induced obesity (DIO) are known to dysregulate the autonomic and metabolic functions related to energy homeostasis throughout the development of obesity, insulin resistance, and ectopic fat accumulation such as hepatic steatosis [5,6]. The effects of HFDs in the brain have emerged as key routes that lead to nutrient oversupply, resulting in the deterioration of metabolic homeostasis. Multiple studies have revealed that long-term HFD consumption leads to neuroinflammation and leptin resistance in the hypothalamus, which governs the food intake response [7,8]. Mechanistically, HFD-mediated endoplasmic reticulum stress appears to trigger apoptosis in hypothalamic neurons [9,10]. Furthermore, clinical and preclinical studies have consistently demonstrated that diets high in saturated fats can impair higher-order brain functions including cognition and psychiatric control [11]. Even short-term HFD intake has been associated with impaired attention and visual memory in human studies [12,13]. Notably, recent evidence has demonstrated that obese people are more prone to developing neurological pathologies such as stroke, depression, and neurodegenerative diseases. Therefore, chronic HFD consumption is considered a risk factor for cognitive impairment, brain aging, and even dementia [14,15,16,17].

Many previous studies have systemically assessed how HFDs impact the genome-wide molecular landscape in peripheral metabolic organs [18,19,20,21]. However, only a few investigations to date have analyzed the impact of a chronic HFD on the brain [22,23]. Given that cells and tissues undergo complex indirect molecular changes over time in response to chronic metabolic stress, characterizing the effects of acute and short-term HFD exposure at the molecular level is particularly critical.

Here, we employed a multiomics approach in mice to identify core differentially expressed molecules and pathways underlying acute HFD metabolic stress. After 10 days of HFD feeding, RNA-seq and LC-MS/MS analyses were respectively conducted to detect differences in the gene expression and metabolite profiles of the whole brain of HFD-exposed and control mice. Intriguingly, we detected alterations in metabolites and transcriptional reprogramming, thus providing insights into the genetic and metabolic changes linked to the central responses to acute HFD stress.

## 2. Materials and Methods

### 2.1. Animals

C57BL/6J male mice were used for the experiments (8–10 weeks old; KRIBB, Daejeon, Republic of Korea). Mice were housed under specific pathogen-free conditions on a 12 h light–dark schedule, and their food and water were provided ad libitum. Animal protocols were performed in accordance with guidelines approved by the Korea Advanced Institute of Science and Technology Animal Care and Use Committee. Mice were fed a normal chow diet (NCD) or high-fat diet (HFD, 60 kcal% fat) for 10 days. After removing the olfactory bulbs and cerebellum, the whole forebrain was separated into two hemispheres. Each hemisphere was subject to RNA-seq and metabolomics analysis.

### 2.2. Total RNA Extraction and RNA-Sequencing

Total RNA was purified using Trizol reagent (Invitrogen, Waltham, MA, USA). RNA quality was evaluated with Agilent 2100 bioanalyzer using the RNA 6000 Nano Chip (Agilent Technologies, Amstelveen, The Netherlands). RNA was quantified using ND-2000 Spectrophotometer (Thermo Inc., Wilmington, DE, USA). RNA libraries were prepared using QuantSeq 3′ mRNA-Seq Library Prep Kit (Lexogen, Inc., Vienna, Austria) according to the manufacturer’s instructions. Five hundred ng of prepared total RNA were hybridized with an oligo-dT primer containing an Illumina-compatible sequence at its 5′ end, followed by reverse transcription. Second strand synthesis was initiated by random primers with an Illumina-compatible linker sequence at the 5′ end. The double-stranded library was purified using magnetic beads. After the library was amplified to add the complete adapter sequences required for cluster generation, it was purified from the PCR components. High-throughput sequencing was performed as single-end 75 bp sequencing using NextSeq 550 (Illumina, Inc., Madison, WI, USA).

QuantSeq 3′ mRNA-Seq reads were aligned using Bowtie2 [24]. Bowtie2 indexes were generated from genome assembly sequences or representative transcript sequences for alignment to the genome and transcriptome. Sequenced reads were trimmed for adaptor sequence, then mapped to mm10 (UCSC) whole genome using Bowtie2. The alignment file was used to assemble transcripts, estimate their abundances and detect differential gene expression. Based on counts from unique and multiple alignments using coverage in Bedtools [25], differentially expressed genes were determined. The RC (read count) data were processed based on the quantile normalization method using EdgeR within R (https://www.R-project.org (accessed on 25 August 2022)) using Bioconductor [26]. Gene classification was based on searches done by DAVID (http://david.abcc.ncifcrf.gov (accessed on 2 September 2022)) and Medline databases (http://www.ncbi.nlm.nih.gov (accessed on 2 September 2022)). Data mining and graphic visualization were performed using ExDEGA (Ebiogen Inc., Seongdong-gu Seoul, Republic of Korea). Sequence data were uploaded in the NCBI GEO (accession number GSE226771).

### 2.3. Global Metabolite Profiling and Data Analysis

Samples were prepared, as previously described at Metabolon, Inc. (Morrisville, NC, USA) [27,28]. Briefly, the MicroLab STAR^®^ system (Hamilton Company, Reno, NV, USA). A recovery standard was added prior to the first step in the extraction process for QC purposes. Proteins were precipitated using methanol for 2 min (Glen Mills GenoGrinder 2000). Extracts were subject to different analyses including UPLC-MS/MS with positive ion mode electrospray ionization, UPLC-MS/MS with negative ion mode electrospray ionization, UPLC-MS/MS polar platform (negative ionization), and GC-MS. Controls were analyzed in parallel with the experimental samples and a pooled matrix sample.

Metabolomic analyses were performed by Metabolon, Inc. using ultrahigh-performance UHPLC/MS/MS, as previously described [29]. The LC/MS part was based on a Waters ACQUITY UPLC and a Thermo Scientific Q-Exactive high resolution/accurate mass spectrometer interfaced with a heated electrospray ionization (HESI-II) source and Orbitrap mass analyzer with 35,000 mass resolution. Extracts were dried and reconstituted in acidic or basic LC-compatible solvents, each of which contained 8 or more injection standards. Sample extracts were analyzed using positive and negative ion conditions using separate columns (Waters UPLC BEH C18-2.1 × 100 mm, 1.7 µm). For elution, water and methanol with 0.1% formic acid or 6.5mM ammonium bicarbonate were used. Samples were also analyzed via negative ionization using a HILIC column (Waters UPLC BEH Amide 2.1 × 150 mm, 1.7 µm) under a gradient consisting of water and acetonitrile with 10 mM ammonium formate. The MS analysis alternated between MS and data dependent MS2 scans using dynamic exclusion, and the scan range was from 80–1000 *m*/*z*. For GC-MS, samples were dried under vacuum for a minimum of 18 h prior to derivatization. Derivatized samples were separated on a 5% diphenyl/95% dimethyl polysiloxane fused silica column (20 m × 0.18 mm ID; 0.18 mm film thickness) with helium as a carrier gas and a temperature ramp from 60 to 340 °C in a 17.5 min period. Samples were analyzed on a Thermo-Finnigan Trace DSQ fast-scanning single-quadrupole mass spectrometer using electron impact ionization. The scan range was from 50–750 *m*/*z*.

Raw data were extracted, peak-identified, and QC processed using Metabolon’s hardware and software. These systems are built on a web-service platform utilizing Microsoft’s. NET technologies, which run on high-performance application servers and fiber-channel storage arrays in clusters to provide active failover and load balancing. Compounds were identified by comparison to library entries of purified standards or recurrent unknown entities Biochemical identifications are based on three criteria: retention index within a narrow RI window of the proposed identification, accurate mass match to the library +/−0.005 amu, and the MS/MS forward and reverse scores between the experimental data and authentic standards. The MS/MS scores are based on a comparison of the ions present in the experimental spectrum to the ions present in the library spectrum. While there may be similarities between these molecules based on one of these factors, the use of all three data points can be utilized to distinguish and differentiate biochemicals. More than 3300 commercially available purified standard compounds have been acquired and registered into LIMS for distribution to both the LC-MS and GC-MS platforms for the determination of their analytical characteristics.

A variety of curation procedures were carried out to ensure that a high-quality data set was made available for statistical analysis and data interpretation. The QC and curation processes were designed to ensure accurate and consistent identification of true chemical entities and to remove those representing system artifacts, misassignments, and background noise. Peaks were quantified using the area under-the-curve. For studies spanning multiple days, a data normalization step was performed to correct variation resulting from instrument interday tuning differences. Essentially, each compound was corrected in run-day blocks by registering the medians to equal one (1.00) and normalizing each data point proportionately. Statistical calculations were made by performing Welch’s two-sample *t*-test and a Wilcoxon test.

## 3. Results

### 3.1. Transcriptome Analysis of the Brain after Short-Term HFD Feeding

**Figure 1:**
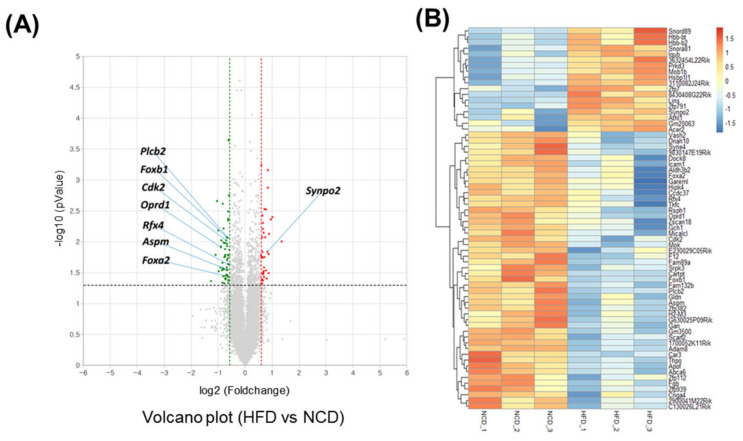
Transcriptome analysis of the brain under short-term HFD feeding. (**A**) Volcano plot analysis of differentially expressed genes (DEGs) after 10 days HFD feeding in which the red and green dot represent fold change > 1.5 and *p* value < 0.05 expression, namely the differential metabolites screened by univariate statistical analysis. (**B**) Heatmap of the significant differential expression gene.

**Figure 2:**
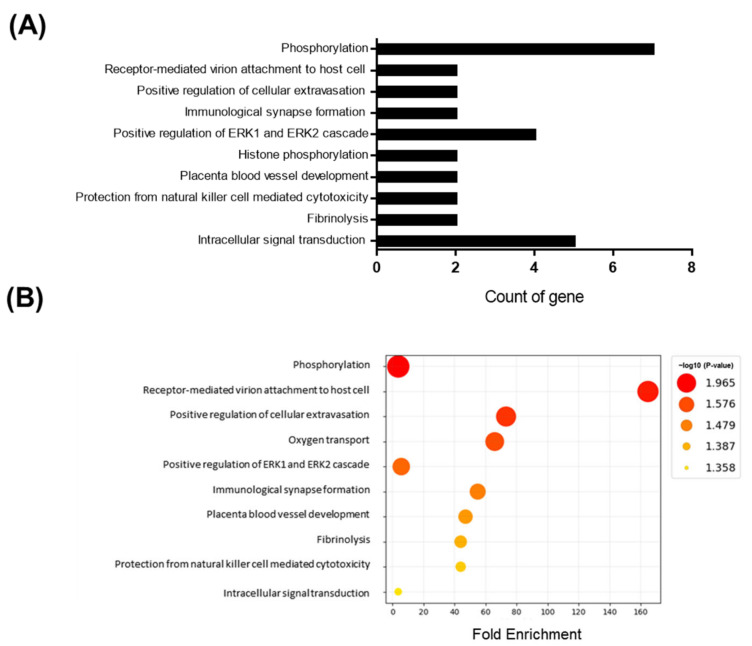
GO enrichment analysis for genes based on DEGs data. Y-axis represents the enriched GO terms; X-axis (**A**) represents the top 10 amount of the genes enriched in GO terms; X-axis (**B**) represents the ratio of the genes enriched in GO terms. The color and size of each bubble represent enrichment significance and the number of genes enriched in a GO term respectively. *p* < 0.05 was used as the threshold to select GO. GO—gene ontology.

Adult male mice were fed an HFD for 10 days. To gain a deeper understanding of the molecular pathways affected by short-term HFD stress in the brain, RNA-Seq analyses were conducted using forebrain tissues. A total of 92 differentially expressed genes (DEGs) (36 upregulated and 56 downregulated) were identified between the control and HFD-exposed animals based on a fold change (FC) threshold of ≥1.5 for upregulation and ≤−1.5 for downregulation were represented with volcano plat (Figure 1A) and heatmap (Figure 1B). All gene expression data are summarized in Supplementary Table S1. Gene ontology (GO) enrichment analysis was conducted to clarify the biological pathways and functions in the mouse brain affected by HFD (Figure 2A). Several modestly but significantly enriched GO categories were identified in the brains of “the HFD-treated mice, including “phosphorylation”, “positive regulation of ERK1 and ERK2 cascade” and “intracellular signal transduction”, suggesting changes in the neural signaling events (Figure 2B). Moreover, KEGG pathway analysis elucidated multiple significantly enriched infection-related pathways (e.g., “African trypanosomiasis”, “human T-cell leukemia virus 1 infection”, “malaria”, “herpes simplex virus 1 infection”), in addition to the “cellular senescence” and “sphingolipid” signaling pathways (Supplementary Figure S1A,B).

### 3.2. Unique Alterations in a Broad Range of Metabolites in the Mouse Brain Exposed to Acute HFD Stress

**Figure 3:**
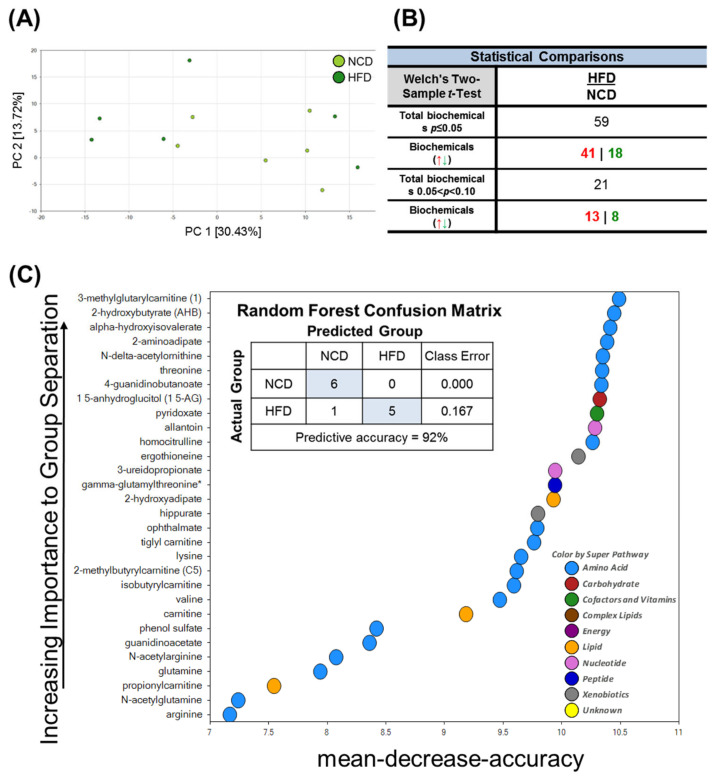
Principal component analysis (PCA), Welch’s two-sample *t*-Test, random forest (RF) of metabolites in the brain in mice fed NCD and HFD. PC is the principal component that distinguishes the NCD group and the HFD group. (**A**) PC1 is the first principal component; PC2 is the second principal component; PC1 explained 30.43% of the variation; PC2 explained 13.72% of the variation. (**B**) Metabolic changes statistical comparisons through Welch’s two-sample *t*-Test between the NCD group and HFD group. 59 statistically significant metabolites (*p* ≤ 0.05) out of 407 were identified in total biochemical. (**C**) Random forest analysis could distinguish between these two groups with a predictive accuracy of 92.0%. The figure presents the 30 top-ranked metabolites and their classification (indicated in the figure, lower right) based on their importance for the identification of the two groups.

To identify HFD-induced metabolic changes, we next performed a global untargeted metabolomics analysis. To identify the differences in the metabolic profiles of the normal chow diet (NCD) and HFD groups, the sample distribution patterns were visualized using a principal components analysis (PCA) score plot. As shown in Figure 3A, our PCA analyses revealed significant heterogeneity and overlap among the NCD and HFD groups. Despite the low degree of separation, Welch’s two-sample *t*-tests revealed 59 statistically significant metabolites (*p* ≤ 0.05) out of 407 identified in total after 10 days of HFD feeding (Figure 3B). These results demonstrated that the brain metabolome was moderately affected when the mice were fed with the HFD. Random forest (RF) analysis was then conducted to identify the most important metabolites and exclude associations by chance (Figure 3C). Using the primary groupings of the HFD and control samples, the RF analysis had a predictive accuracy of 92%. The main metabolites that were dysregulated in the HFD brain tissue samples compared to the NCD (control) samples were involved in oxidative stress, amino acid metabolism (e.g., branched-chain amino acid catabolism, lysine metabolism), and microbiome metabolism.

### 3.3. HFD Treatment Increases Oxidative Stress

**Figure 4:**
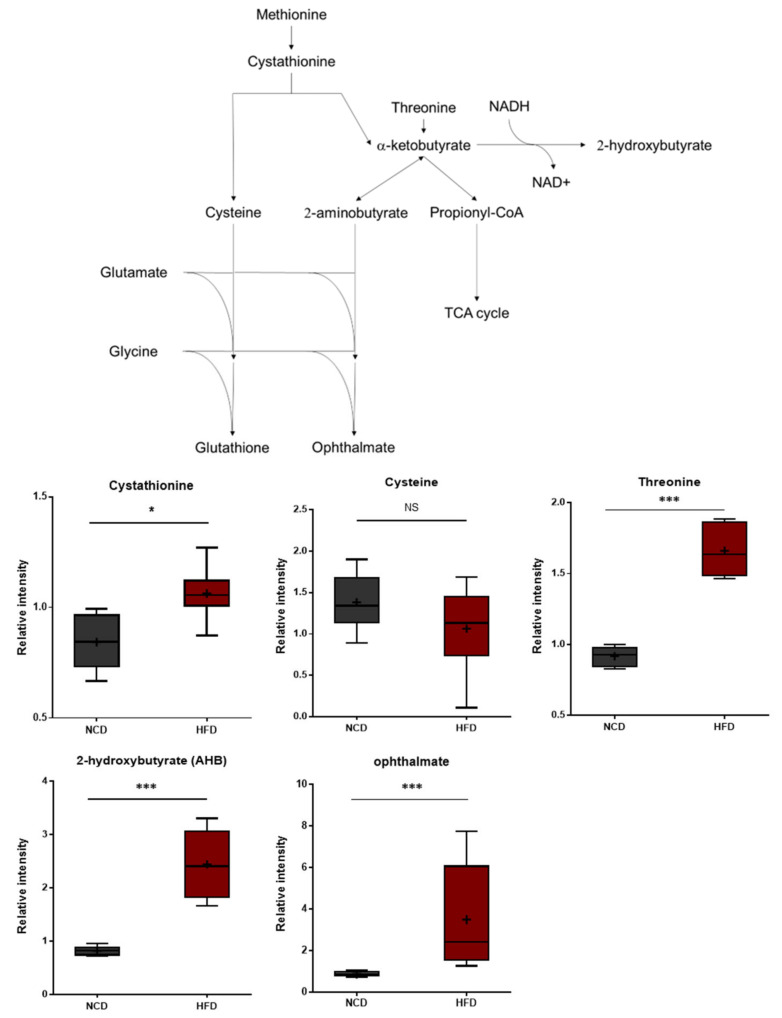
Pathway statistically significant biochemical profiled between NCD group and HFD group. In methionine, the cystenine–glutathione metabolism pathway leads to AHB and ophthalmate production increased in the HFD group which shows increased oxidative stress within cells. Data were presented as mean ± SD (*n* = 6) and two-way ANOVA with Bonferroni’s multiple comparison test. * *p* < 0.05; *** *p* < 0.001.

Compared to the control group, the HFD group exhibited significant differences in 2-hydroxybutyrate (AHB) and ophthalmate (Figure 4). These metabolites are derived from α-ketobutyrate, a metabolite that can be formed via cystathionine in the transsulfuration pathway or from threonine. AHB is synthesized when α-ketobutyrate is reduced by lactate dehydrogenase (LDH) or α-hydroxybutyrate dehydrogenase (alpha-HBDH) [30]. Notably, AHB has also been identified as an early marker for insulin resistance and altered glucose tolerance [31,32]. Changes in AHB levels may thus be linked to alterations in glucose sensitivity in HFD-fed mice [33]. Ophthalmate, on the other hand, is a compositional derivative of glutathione that is formed when the cysteine residue of glutathione is replaced with a 2-aminobutyrate residue [34,35,36]. A 2-aminobutyrate can be derived enzymatically from α-ketobutyrate. The levels of AHB and ophthalmate typically increase in response to increased oxidative stress within cells. The metabolic changes described herein are therefore consistent with increases in oxidative stress.

### 3.4. Elevated Levels of Oxidized Polyunsaturated Fatty Acids

**Figure 5:**
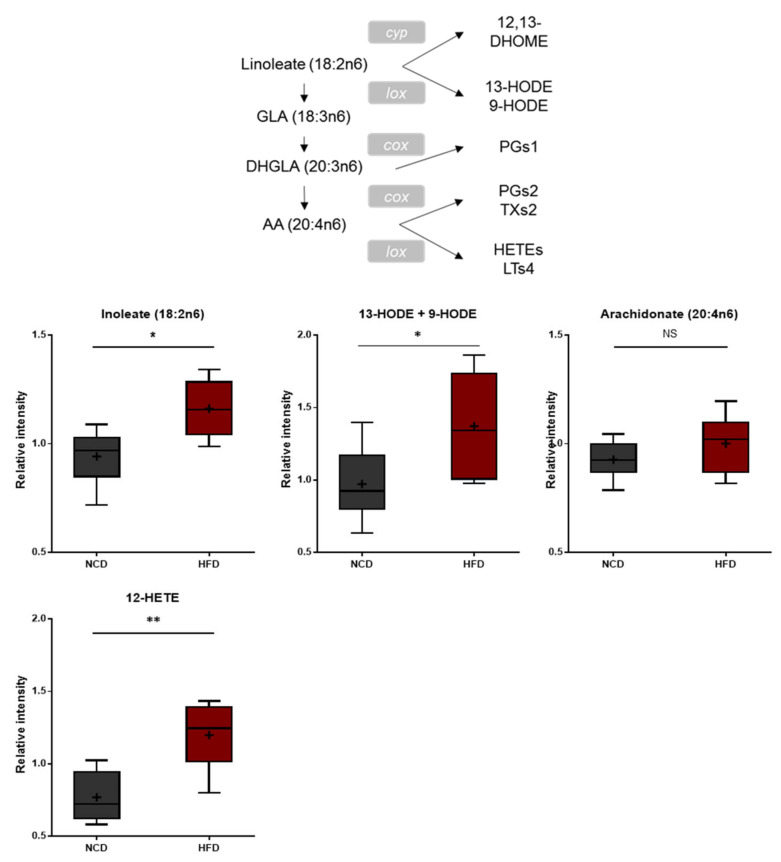
Pathway of statistically significant biochemicals profiled between NCD group and HFD group. In fatty acid, monohydroxy metabolism pathway markers of 13-HODE, + 9-HODE, and 12-HETE were elevated in the HFD group which suggests increased oxidative stress and inflammation. Data were presented as mean ± SD (*n* = 6) and two-way ANOVA with Bonferroni’s multiple comparison test. * *p* < 0.05; ** *p* < 0.01.

Polyunsaturated fatty acids (PUFAs) ingested from the diet or produced from phospholipase A-mediated hydrolysis are peroxidized enzymatically by lipoxygenases or nonenzymatically through reactive oxygen radicals [37,38]. These bioactive oxidized PUFAs have been linked to a wide variety of pathological conditions such as cardiovascular disease, diabetes, inflammation, obesity, and cancer [39,40,41,42,43]. The brain tissues of the HFD-fed mice displayed elevated levels of 13-hydroxyoctadecadienoic acid (13-HODE), 9-hydroxyoctadecadienoic acid (9-HODE), and 12-hydroxyeicosatetraenoic acid (12-HETE) (Figure 5), suggesting increased oxidative stress and inflammation.

### 3.5. Changes in Branched Amino Acid (BCAA) Metabolism

**Figure 6:**
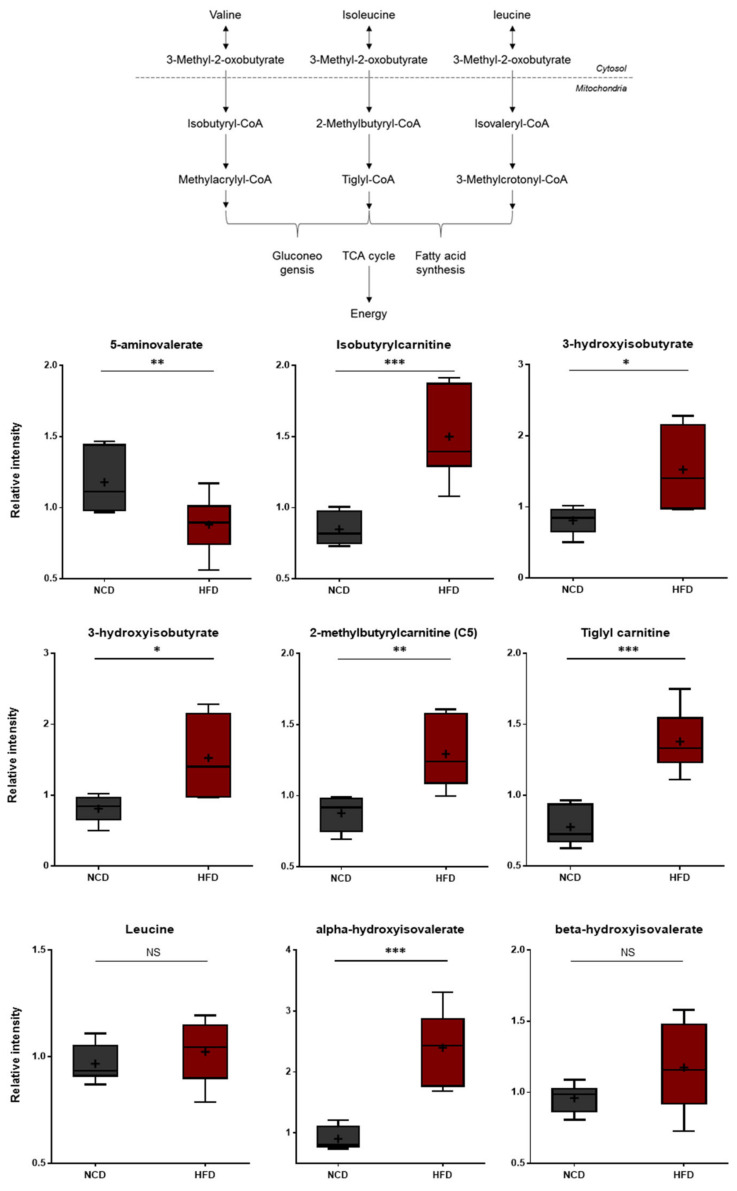
Pathway of statistically significant biochemicals profiled between NCD group and HFD group. Valine/Isoleucine/Leucine-Catabolites increased which present branched-chain amino acid metabolism is altered in the HFD group. Data were presented as mean ± SD (*n* = 6) and two-way ANOVA with Bonferroni’s multiple comparison test. * *p* < 0.05; ** *p* < 0.01; *** *p* < 0.001.

The HFD and control NCD groups exhibited clear differences in BCAA metabolism. As shown in Figure 6, the HFD group presented higher levels of isoleucine and valine. Additionally, the HFD group accumulated higher levels of several BCAA catabolites, including alpha-hydroxyisovalerate, beta-hydroxyisovalerate, 2-methylbutrylcarnitine, tiglyl carnitine, isobutyrylcarnitine, and 3-hydroxyisobutyrlcarnitine. Alpha-hydroxyisovalerate is a 2-hydroxycarboxylic acid formed from 4-methyl-2-oxopentanoic acid (i.e., a keto acid derived from leucine). On the other hand, 2-methylbutryl-carnitine, tiglyl carnitine, isobutyrylcarnitine, and 3-hydroxy-isobutyrlcarnitine are carnitine derivatives of fatty acid coenzyme A (CoA), which are formed in the downstream steps of BCAA catabolism. Collectively, these changes are consistent with alterations in the rates of BCAA utilization and/or uptake in the HFD-fed mice, suggesting alterations in energy metabolism and neurotransmitter synthesis [44].

### 3.6. Changes in Lysine Metabolism

**Figure 7:**
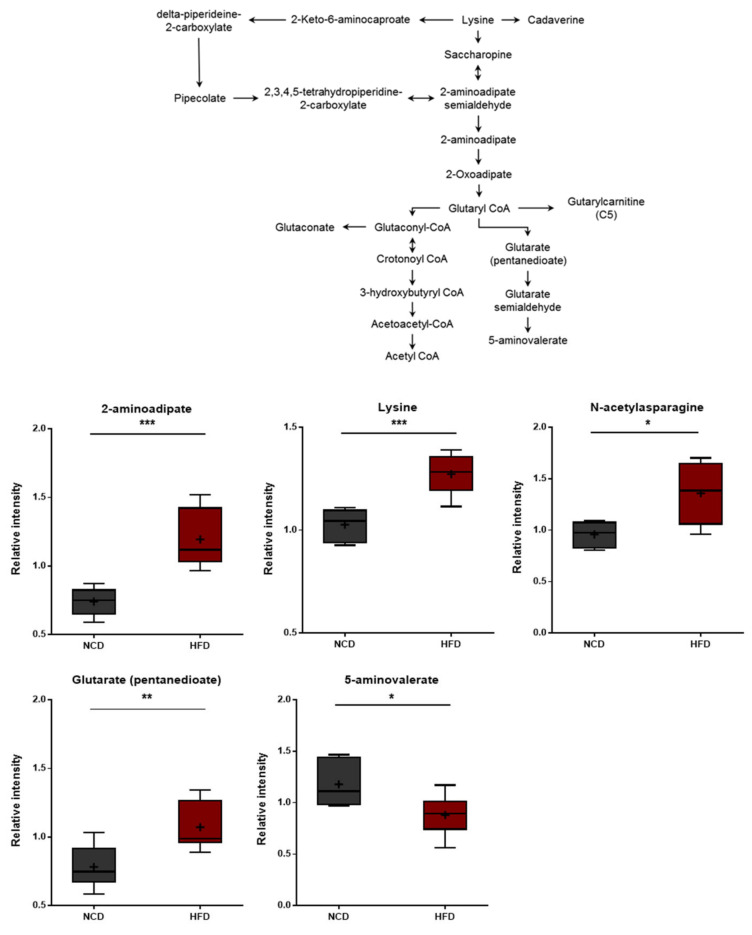
Pathway of statistically significant biochemicals profiled between NCD group and HFD group. Metabolites involved in the lysine metabolism were altered in the HFD group. Data were presented as mean ± SD (*n* = 6) and two-way ANOVA with Bonferroni’s multiple comparison test. * *p* < 0.05; ** *p* < 0.01; *** *p* < 0.001.

Lysine degradation in humans occurs through two distinct but convergent pathways: the saccharopine pathway and the pipecolate pathway. The saccharopine pathway is the main catabolic pathway through which lysine catabolism occurs. However, the pipecolate pathway is dominant in the brain [45]. Higher levels of lysine and several lysine-derived catabolites (pipecolate, 2-aminoadipate, and glutarate) were detected in the HFD group (Figure 7). In turn, these changes were consistent with alterations in the rates of lysine uptake and/or catabolism. It should be noted that 2-aminoadipate is a marker of oxidative stress [46]. The 2-Aminoadipate can also antagonize neuroexcitatory activity modulated by the N-methyl-D-aspartate (NMDA) receptor [47]. Moreover, lower levels of 5-aminovalerate were observed in the HFD group, and 5-Aminovalerate can be generated either endogenously or through bacterial activity. Lower levels of this metabolite may thus reflect alterations in gut microbiome metabolism, as discussed below [48]. The HFD-fed group displayed elevated levels of all three branched-chain amino acids (BCAAs), including leucine, isoleucine, and valine (Figure 6). The changes in these amino acids reflect either increased protein breakdown within brain cells (e.g., neurons, glia) or increased uptake of amino acids into the brain from the bloodstream. Among other amino acid metabolites, significant increases in the lysine catabolite 5-aminovalerate were also found in the brain tissues of the HFD-fed mice, indicating alterations in the utilization, catabolism, and/or uptake of lysine.

### 3.7. N-Acetylamino Acids

**Figure 8:**
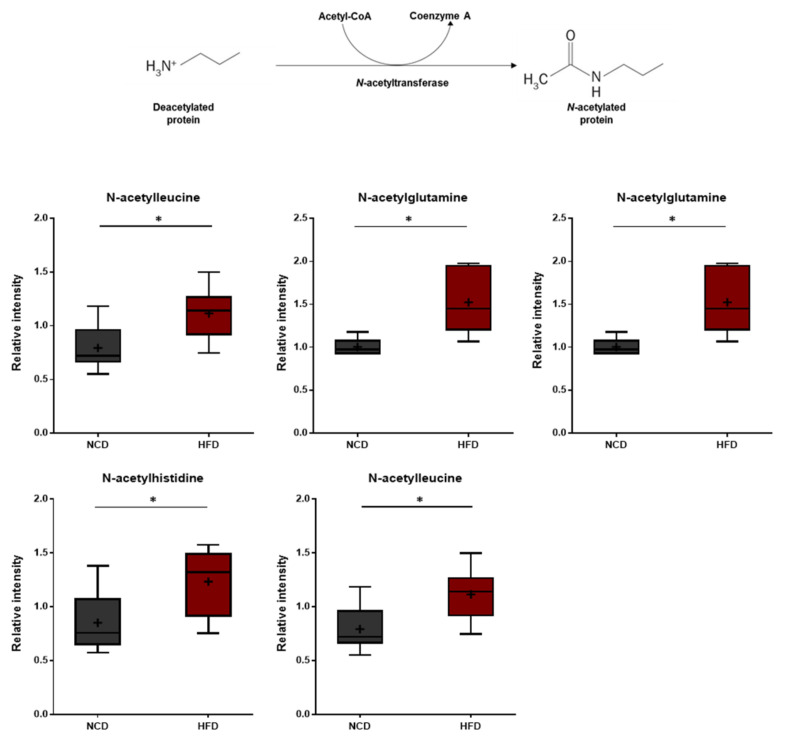
Pathway of statistically significant biochemicals profiled between NCD group and HFD group. Elevated N-terminal acetyl amino acids were detected in the HFD group. Data were presented as mean ± SD (*n* = 6) and two-way ANOVA with Bonferroni’s multiple comparison test. * *p* < 0.05.

N-acetyl amino acids are synthesized either via specific N-acetyltransferases or throughout the degradation of N-acetylated proteins by specific hydrolases. N-terminal acetylation of proteins is known to protect and stabilize proteins [49,50]. In this study, the HFD group displayed elevated levels of several N-acetylated amino acids including N-acetylthreonine, N-acetylasparagine, N-acetylglutamine, N-acetylhistidine, and N-acetylleucine (Figure 8). These changes suggest that the HFD treatment induces amino acid imbalances such as increased protein breakdown.

### 3.8. Alterations in Arginine Metabolism, Urea Cycle Metabolites, and Uracil Metabolites

**Figure 9:**
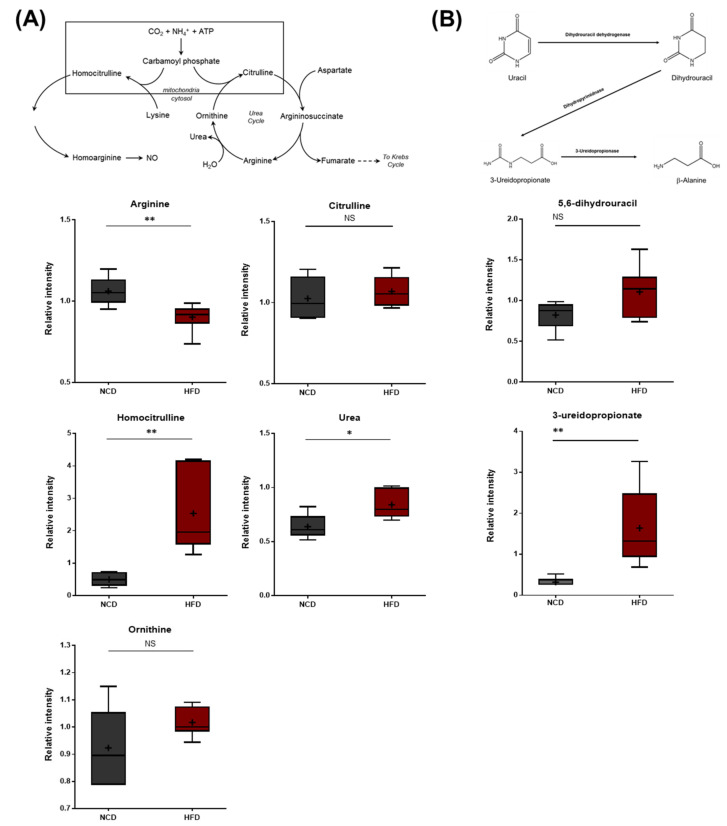
Pathway of statistically significant biochemicals profiled between NCD group and HFD group. Metabolites in (**A**) arginine metabolism, urea cycle pathway, and (**B**) uracil catabolism were altered in the HFD group. Urea was found at an elevated level via homocitrulline. Also, the HFD group exhibited elevated levels of uracil catabolism. Data were presented as mean ± SD (*n* = 6) and two-way ANOVA with Bonferroni’s multiple comparison test * *p* < 0.05; ** *p* < 0.01.

The HFD group exhibited lower arginine levels, which were accompanied by elevated urea levels (Figure 9A), suggesting increased arginase activity that hydrolyzes arginine into urea and ornithine. In addition to these changes, the HFD group also displayed elevated levels of homocitrulline, a metabolite formed from lysine and carbamoyl phosphate via the activity of ornithine-transcarbamylase (OTC). Homocitrulline is a metabolic precursor of homoarginine, an arginine analog that can inhibit arginase. It was unclear whether homocitrulline accumulated due to changes in lysine metabolism (described above) or alterations in arginase activity. N-delta-acetylornithine was also present at lower levels in the HFD group. This metabolite can be derived from both ornithine and glutamate. The HFD brain samples also exhibited elevated levels of 5,6-dihydrouracil and 3-ureidopropionate, both of which are metabolites involved in uracil catabolism (Figure 9B). These changes were consistent with alterations in the rate of uracil degradation and may be related to changes in the urea cycle, as both involve changes in nitrogen balance.

### 3.9. Gut Microbiome Metabolites

**Figure 10:**
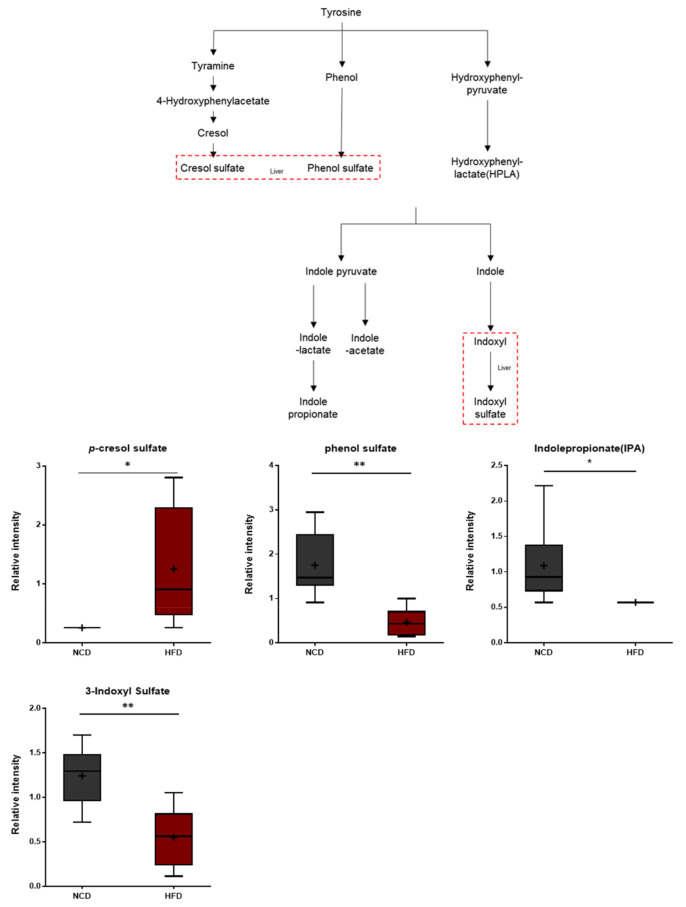
Pathway of statistically significant biochemicals profiled between NCD group and HFD group. Gut microbiome-derived metabolites such as *p*-cresol sulfate, phenol sulfate, indole propionate, and 3-indoxyl sulfate were altered in the HFD group. Data were presented as mean ± SD (*n* = 6) and two-way ANOVA with Bonferroni’s multiple comparison test. * *p* < 0.05; ** *p* < 0.01.

The brains of the HFD-fed mice exhibited alterations in the levels of ippurate and several aromatic amino acid catabolites of microbial origin such as *p*-cresol sulfate, phenol sulfate, indolepropionate, and 3-indoxyl sulfate (Figure 10). Hippurate is synthesized in the liver from glycine and benzoic acid [51]. The *p*-cresol sulfate and phenol sulfate are generated from the microbial breakdown of tyrosine, whereas indolepropionate and 3-indoxyl sulfate are formed via the microbial breakdown of tryptophan. Therefore, the observed alterations in the levels of these metabolites in the brain could reflect differences in the rates at which these catabolites are produced or, alternatively, differences in the rates of brain uptake.

### 3.10. Integration Analysis of Transcriptomic and Metabolomic Data

**Figure 11:**
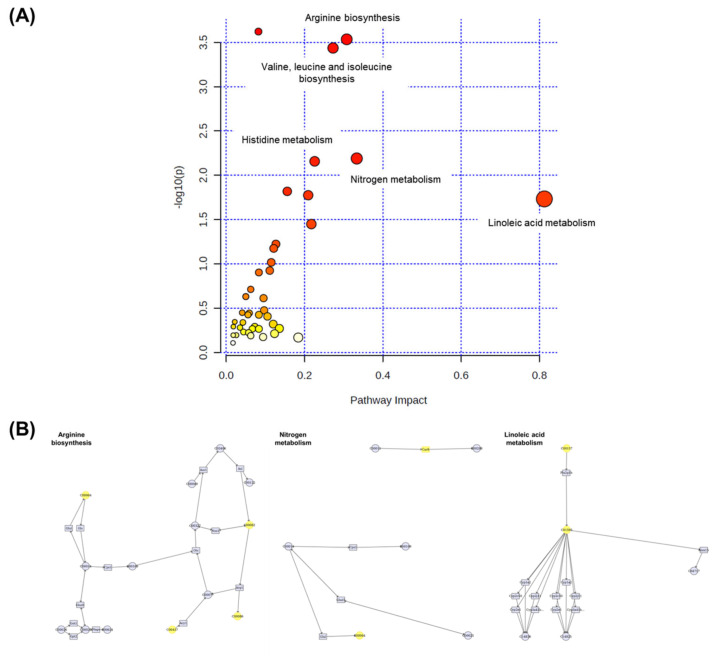
The pathway impact analysis. (**A**) The metabolic pathways were represented as circles according to their scores from enrichment (y-axis) and topology analyses (pathway impact and x-axis) using MetaboAnalyst 5.0. Darker circle colors indicated more significant changes in metabolites in the corresponding pathway. The size of the circle corresponds to the pathway impact score and was correlated with the centrality of the involved metabolites. (**B**) Three most representative pathways from metabolic pathway changes. The yellow circle represents the affected part of the detailed pathway.

Multiomic profiling is a comprehensive approach that provides more indepth insights into the molecular mechanisms of biological phenomena compared to transcriptomic, proteomic, or metabolomic analyses alone [52]. Therefore, MetaboAnalyst 5.0 was used in this study to conduct joint pathway analysis of transcriptomic and metabolomic data. Notably, the dysregulated pathways at both the metabolomic and mRNA expression levels in acute HFD-exposed brain were involved in aminoacyl-tRNA biosynthesis, arginine biosynthesis, valine–leucine–isoleucine biosynthesis, and nitrogen metabolism, among other processes. Figure 11 shows the altered pathways, including the metabolic pathways of interest. Four metabolic pathways (i.e., aminoacyl-tRNA biosynthesis, arginine biosynthesis, valine–leucine–isoleucine biosynthesis, and nitrogen metabolism) had *p*-values < 0.05 and impact coefficients > 0.2, indicating that these pathways were regulated due to HFD treatment.

## 4. Discussion

High-fat diets are known to contribute to central energy homeostasis and neural functions by dysregulating synaptic plasticity, exacerbating neuroinflammation, and suppressing long-term potentiation. Although many studies have demonstrated that chronic HFD treatment induces gene expression changes in the brain, the impact of short-term HFD on the brain had remained largely unexplored. Therefore, this study sought to define the major molecular changes in the brain induced by acute HFD treatment at the transcriptome and metabolite level in an unbiased manner.

According to our RNA-Seq results, the expression of 92 mRNAs changed significantly in the mouse brain in response to acute HFD exposure, of which 36 mRNAs were upregulated and 56 were downregulated. GO enrichment analysis of the differentially expressed mRNAs revealed the dysregulation of the “phosphorylation”, “positive regulation of ERK1 and ERK2 cascade”, and “intracellular signal transduction” pathways, among others. A recent transcriptomic study conducted by Yoon et al. demonstrated that chronic (i.e., eight weeks) HFD treatment altered the expression of synaptotagmin genes and NMDA receptor subunits in mice [22]. In our 10 day study, the HFD-treated mice did not exhibit perturbations in the aforementioned target genes. Instead, we observed aberrant upregulation of serotonin receptor 2A and synaptopodin-2, which is known to control dendritic spine plasticity in the hippocampus [53]. Moreover, we observed a notable downregulation of several key signaling factors involved in neural communication such as phospholipase C 1 beta, opioid receptor delta 1, and cyclin-dependent kinase 2 (Cdk2). Acute HFD treatment also markedly affected the expression of genes responsible for brain development and morphogenesis (e.g., Foxb1, Foxa2, ASPM, RFX4) [54,55,56,57], further confirming the link between acute HFD treatment and neuronal signaling defects.

Surprisingly, acute HFD treatment had no effect on metabolic endocrine factors or enzymes associated with energy metabolism. Instead, we observed changes in the expression of metabolic enzymes such as aldehyde dehydrogenase (ALDH3b2), which is involved in the metabolism of a toxic aldehydic metabolite of dopamine in the brain [58], suggesting possible changes in catecholamine networks. Our findings also demonstrated the downregulation of GTP cyclohydrolase 1 (GCH1), a known Parkinson’s disease (PD) risk gene. Specifically, GCH1 mediates the rate-limiting step for the production of tetrahydrobiopterin (BH4), an essential cofactor required for the synthesis of monoaminergic neurotransmitters such as serotonin and dopamine [59]. GCH1 deficiency was recently shown to activate the innate immune response in the brain [60]. Furthermore, genes involved in immunity and infection [e.g., chemokine ligand 19 (ccl19), dedicator of cytokinesis 8 (DOCK8), and intercellular adhesion molecule-1 (ICAM-1)] [61,62] were also significantly dysregulated. Taken together, these transcriptomic changes in the brain in response to acute HFD feeding provide key insights into the molecular mechanisms underlying HFD-induced neural dysfunction, which can lead to impairments in cognition, mood control, and central nervous system (CNS) immunity.

Consistent with the results of our RNA-seq analyses, acute HFD treatment did not alter the levels of metabolites related to energy and glucose metabolism. Instead, our metabolomic data revealed marked HFD-induced changes in metabolites related to oxidative stress, amino acid metabolism (e.g., BCAA catabolism and lysine metabolism), nitrogen metabolism (e.g., urea cycle and uracil metabolism), and the gut microbiome. In a previous study, nuclear magnetic resonance (NMR)-based metabolomics analyses revealed significant changes in alanine, creatine/phosphocreatine, taurine, and myo-inositol levels after eight weeks of chronic HFD feeding [63]. In another study, long-term HFD treatment modulated the levels of membrane phospholipids and diacylglycerol in the brain [64]. Although both acute and chronic HFD treatment increased the levels of metabolites associated with oxidative stress, acute HFD treatment appeared to have a more pronounced effect on amino acid metabolism and nitrogen metabolism in the brain during the early stages of the feeding experiment. The major changes in BCAA and lysine metabolism observed after HFD treatment suggested that HFD could significantly affect neural control. Glutamate, which is synthesized via BCAA transamination, is an excitatory neurotransmitter and substrate for the production of the major inhibitory neurotransmitter gamma-aminobutyric acid (GABA). Current understandings of the role of BCAAs in the brain are consistent with the association of glutamatergic and/or GABAergic systems in the etiology of neurological disorders. Fluctuations in BCAA levels significantly influence CNS function, particularly the balance between excitation and inhibition. BCAA metabolism contributes to the synthesis of new glutamate when this amino acid becomes depleted in the neurons in response to oxidative stress, and, therefore, this process plays a crucial role in brain function homeostasis. Decreases in the levels of the BCAAs are typically observed in psychiatric patients with major depressive disorder, immune-related major depression, and bipolar disorder [65,66,67]. BCAA levels are also elevated in the hippocampus after antidepressant treatment [68]. Furthermore, 5-aminovaleric acid, which is endogenously synthesized or derived from the metabolism of lysine by gut microbiota, is also known to act as a methylene homolog of GABA and functions as a weak GABA agonist [69]. Recently, 5-aminovaleric acid was also identified in the plasma and brain tissues of Alzheimer’s disease patients [70].

The elevated levels of N-acetyl amino acids (e.g., N-acetylthreonine, N-acetylasparagine, N-acetylglutamine, N-acetylhistidine, and N-acetylleucine) in response to acute HFD also drew our attention. N-terminal-acetylation reactions mainly occur through N-acetyltransferase enzymes (NATs) [49,50]. N-acetylated amino acids such as N-acetylhistidine can be also produced by N-acylpeptide hydrolases which can degrade target proteins. In addition to the NAT enzymes and protein-based acetylation, Free amino acids can be acetylated. For example, N-acetylhistidine can be biosynthesized from L-histidine and acetyl-CoA by the enzyme histidine N-acetyltransferase. Many N-acetylamino acids are classified as uremic toxins when they are found in high abundance in the serum or plasma [71]. Uremic toxins are a diverse group of endogenously produced metabolites that can cause kidney damage and neurological deficits if not properly cleared or eliminated by the kidneys. In addition to precisely defining the molecular pathways through which HFD feeding leads to increased N-acetylamino acids, their effects on neural functions should also be further investigated.

A nearly fivefold increase in the levels of *p*-cresol sulfate (PCS), which is produced by gut microbiota from tyrosine degradation, was among the most striking changes induced by acute HFD treatment. PCS and 3-indoxyl sulfate are classified as uremic toxins that may contribute to CNS toxicity [72]. Moreover, growing evidence has demonstrated that PCS triggers cell death and dysfunction by inducing oxidative stress and inflammation, impairing mitochondrial dynamics, and suggesting pathogenic roles of PCS in CNS diseases [72,73]. Elevated levels of PCS have been detected in the urine and feces of autistic patients [74,75,76]. In rodents, PCS administration induced autism-like behavioral changes [77,78,79]. Additionally, acute HFD lowered indole proprionate (IPA), another microbiota-derived metabolite of tryptophan. As a strong free radical scavenger, IPA is known to play a neuroprotective role in lowering inflammation, lipid oxidation, and free radical formations [80]. Moreover, IPA levels significantly decrease in type two diabetes and obesity [81,82]. Collectively, our findings demonstrated that HFD administration for 10 days likely remodeled the gut microbiome and decreased intestinal epithelial permeability, thereby inducing dynamic changes in gut-derived metabolites that aggravate brain homeostasis and neural activities.

In conclusion, our study provides a detailed characterization of the molecular changes that occur in the brain in response to acute HFD treatment. Future studies will need to investigate the region-specific changes in the brain using various omics approaches such as epigenomics and proteomics. Single-cell data with a higher temporal resolution will also be required to identify reliable HFD biomarkers and gain mechanistic insights into whether such molecular dysregulations could be associated with obesity-linked central homeostasis and related psychiatric disorders.

## Data Availability

Data available in a publicly accessible repository.

## Supplementary Materials

The following supporting information can be downloaded at: https://www.mdpi.com/article/10.3390/metabo13030407/s1, Figure S1: KEGG pathway enrichment analysis for genes based on DEGs data; Figure S2: Principal component analysis (PCA) of RNA-seq result in brain in mouse fed NCD and HFD; Table S1: List of significant upregulated genes of RNA-Seq differential gene expression analysis; Table S2: List of significant downregulated genes of RNA-Seq differential gene expression analysis; Table S3: Summary of mapping rate of RNA-seq on DEGs data; Table S4: Summary of joint pathway integration analysis of transcriptomic and metabolomic data with Metaboanalyst 5.0; Table S5: Normality test result of 59 selected compounds of metabolon data; Table S6: Summary of Welch’s *t*-test and Wilcoxon test *p*-value and *q*-value result of 59 selected compounds of metabolon data. RNA-seq QC files.
