# Supplementary material for: Metabolic and Transcriptomic Changes in the Mouse Brain in Response to Short-Term High-Fat Metabolic Stress
Source: Metabolites. 2023 Mar 9;13(3):407. doi: 10.3390/metabo13030407 (PMC10051449; doi:10.3390/metabo13030407)
Supplement: Supplementary file 1 [file metabolites-13-00407-s001.zip › 230207_Metabolites_FastQC/HFD_1_fastqc.html]

HFD\_1.fastq.gz FastQC Report 

FastQC Report

월 22 8월 2022  
HFD\_1.fastq.gz

## Summary

- Basic Statistics
- Per base sequence quality
- Per tile sequence quality
- Per sequence quality scores
- Per base sequence content
- Per sequence GC content
- Per base N content
- Sequence Length Distribution
- Sequence Duplication Levels
- Overrepresented sequences
- Adapter Content

## Basic Statistics

| Measure | Value |
| --- | --- |
| Filename | HFD\_1.fastq.gz |
| File type | Conventional base calls |
| Encoding | Sanger / Illumina 1.9 |
| Total Sequences | 16810127 |
| Sequences flagged as poor quality | 0 |
| Sequence length | 76 |
| %GC | 45 |

## Per base sequence quality

## Per tile sequence quality

## Per sequence quality scores

## Per base sequence content

## Per sequence GC content

## Per base N content

## Sequence Length Distribution

## Sequence Duplication Levels

## Overrepresented sequences

| Sequence | Count | Percentage | Possible Source |
| --- | --- | --- | --- |
| GGGTTGGGGATTTAGCTCAGTGGTAGAGCGCTTGCCTAGCAAGCGCAAGG | 306099 | 1.8209202107753262 | No Hit |
| GGTTGGGGATTTAGCTCAGTGGTAGAGCGCTTGCCTAGCAAGCGCAAGGC | 252752 | 1.5035698421552675 | No Hit |
| GGGGTTGGGGATTTAGCTCAGTGGTAGAGCGCTTGCCTAGCAAGCGCAAG | 239581 | 1.4252182627769558 | No Hit |
| TTGGGGATTTAGCTCAGTGGTAGAGCGCTTGCCTAGCAAGCGCAAGGCCC | 195648 | 1.1638698505966076 | No Hit |
| GTTGGGGATTTAGCTCAGTGGTAGAGCGCTTGCCTAGCAAGCGCAAGGCC | 155155 | 0.9229852933294317 | No Hit |
| GGGGATTTAGCTCAGTGGTAGAGCGCTTGCCTAGCAAGCGCAAGGCCCTG | 103797 | 0.617467078029809 | No Hit |
| TGGGGATTTAGCTCAGTGGTAGAGCGCTTGCCTAGCAAGCGCAAGGCCCT | 85303 | 0.5074500626913765 | No Hit |
| TGGGGTTGGGGATTTAGCTCAGTGGTAGAGCGCTTGCCTAGCAAGCGCAA | 57780 | 0.3437213770008995 | No Hit |
| GGGATTTAGCTCAGTGGTAGAGCGCTTGCCTAGCAAGCGCAAGGCCCTGG | 55903 | 0.33255548872414825 | No Hit |
| AGGGTTGGGGATTTAGCTCAGTGGTAGAGCGCTTGCCTAGCAAGCGCAAG | 41105 | 0.24452521982731004 | No Hit |
| TGGGTTGGGGATTTAGCTCAGTGGTAGAGCGCTTGCCTAGCAAGCGCAAG | 37130 | 0.22087875957153683 | No Hit |
| AGGGGTTGGGGATTTAGCTCAGTGGTAGAGCGCTTGCCTAGCAAGCGCAA | 36457 | 0.21687522051439587 | No Hit |
| GGATTTAGCTCAGTGGTAGAGCGCTTGCCTAGCAAGCGCAAGGCCCTGGG | 33707 | 0.20051603417392386 | No Hit |
| GGGGTGGGGATTTAGCTCAGTGGTAGAGCGCTTGCCTAGCAAGCGCAAGG | 28106 | 0.167196833194657 | No Hit |
| GGGTGGGGATTTAGCTCAGTGGTAGAGCGCTTGCCTAGCAAGCGCAAGGC | 25630 | 0.15246761669319928 | No Hit |
| AGGTTGGGGATTTAGCTCAGTGGTAGAGCGCTTGCCTAGCAAGCGCAAGG | 24089 | 0.14330052354750206 | No Hit |
| TGGTTGGGGATTTAGCTCAGTGGTAGAGCGCTTGCCTAGCAAGCGCAAGG | 21855 | 0.13001091544400586 | No Hit |
| GCTCAGTGGTAGAGCGCTTGCCTAGCAAGCGCAAGGCCCTGGGTTCGGTC | 20828 | 0.12390150294521868 | No Hit |
| GATTTAGCTCAGTGGTAGAGCGCTTGCCTAGCAAGCGCAAGGCCCTGGGT | 19428 | 0.1155731898991602 | No Hit |
| CGGGGTTGGGGATTTAGCTCAGTGGTAGAGCGCTTGCCTAGCAAGCGCAA | 18035 | 0.10728651841833199 | No Hit |
| CCAACCCTCACACACACGAGAACTAACACTAATAGCCCTTCACATAATTC | 17356 | 0.10324728659099364 | No Hit |

## Adapter Content

Produced by FastQC (version 0.11.8)
