# Supplementary material for: Metabolic and Transcriptomic Changes in the Mouse Brain in Response to Short-Term High-Fat Metabolic Stress
Source: Metabolites. 2023 Mar 9;13(3):407. doi: 10.3390/metabo13030407 (PMC10051449; doi:10.3390/metabo13030407)
Supplement: Supplementary file 1 [file metabolites-13-00407-s001.zip › 230207_Metabolites_FastQC/HFD_2_fastqc.html]

HFD\_2.fastq.gz FastQC Report 

FastQC Report

월 22 8월 2022  
HFD\_2.fastq.gz

## Summary

- Basic Statistics
- Per base sequence quality
- Per tile sequence quality
- Per sequence quality scores
- Per base sequence content
- Per sequence GC content
- Per base N content
- Sequence Length Distribution
- Sequence Duplication Levels
- Overrepresented sequences
- Adapter Content

## Basic Statistics

| Measure | Value |
| --- | --- |
| Filename | HFD\_2.fastq.gz |
| File type | Conventional base calls |
| Encoding | Sanger / Illumina 1.9 |
| Total Sequences | 21676101 |
| Sequences flagged as poor quality | 0 |
| Sequence length | 76 |
| %GC | 44 |

## Per base sequence quality

## Per tile sequence quality

## Per sequence quality scores

## Per base sequence content

## Per sequence GC content

## Per base N content

## Sequence Length Distribution

## Sequence Duplication Levels

## Overrepresented sequences

| Sequence | Count | Percentage | Possible Source |
| --- | --- | --- | --- |
| GGGTTGGGGATTTAGCTCAGTGGTAGAGCGCTTGCCTAGCAAGCGCAAGG | 367004 | 1.6931273756290395 | No Hit |
| GGTTGGGGATTTAGCTCAGTGGTAGAGCGCTTGCCTAGCAAGCGCAAGGC | 304838 | 1.4063322550490054 | No Hit |
| GGGGTTGGGGATTTAGCTCAGTGGTAGAGCGCTTGCCTAGCAAGCGCAAG | 278244 | 1.2836441387683144 | No Hit |
| TTGGGGATTTAGCTCAGTGGTAGAGCGCTTGCCTAGCAAGCGCAAGGCCC | 248768 | 1.147660273404336 | No Hit |
| GTTGGGGATTTAGCTCAGTGGTAGAGCGCTTGCCTAGCAAGCGCAAGGCC | 193878 | 0.8944320752150028 | No Hit |
| GGGGATTTAGCTCAGTGGTAGAGCGCTTGCCTAGCAAGCGCAAGGCCCTG | 137250 | 0.6331858298685727 | No Hit |
| TGGGGATTTAGCTCAGTGGTAGAGCGCTTGCCTAGCAAGCGCAAGGCCCT | 103190 | 0.4760542497933553 | No Hit |
| GGGATTTAGCTCAGTGGTAGAGCGCTTGCCTAGCAAGCGCAAGGCCCTGG | 76985 | 0.3551607367026016 | No Hit |
| TGGGGTTGGGGATTTAGCTCAGTGGTAGAGCGCTTGCCTAGCAAGCGCAA | 62522 | 0.28843748236825434 | No Hit |
| AGGGTTGGGGATTTAGCTCAGTGGTAGAGCGCTTGCCTAGCAAGCGCAAG | 47620 | 0.2196889560534895 | No Hit |
| GGATTTAGCTCAGTGGTAGAGCGCTTGCCTAGCAAGCGCAAGGCCCTGGG | 46079 | 0.21257974393088497 | No Hit |
| AGGGGTTGGGGATTTAGCTCAGTGGTAGAGCGCTTGCCTAGCAAGCGCAA | 42517 | 0.19614689929706455 | No Hit |
| TGGGTTGGGGATTTAGCTCAGTGGTAGAGCGCTTGCCTAGCAAGCGCAAG | 41546 | 0.19166731138593607 | No Hit |
| GGGGTGGGGATTTAGCTCAGTGGTAGAGCGCTTGCCTAGCAAGCGCAAGG | 29927 | 0.1380644978541113 | No Hit |
| GCTCAGTGGTAGAGCGCTTGCCTAGCAAGCGCAAGGCCCTGGGTTCGGTC | 28717 | 0.13248231312448674 | No Hit |
| AGGTTGGGGATTTAGCTCAGTGGTAGAGCGCTTGCCTAGCAAGCGCAAGG | 28675 | 0.13228855134048323 | No Hit |
| GGGTGGGGATTTAGCTCAGTGGTAGAGCGCTTGCCTAGCAAGCGCAAGGC | 27924 | 0.1288239061074683 | No Hit |
| GATTTAGCTCAGTGGTAGAGCGCTTGCCTAGCAAGCGCAAGGCCCTGGGT | 26067 | 0.12025686722902794 | No Hit |
| TGGTTGGGGATTTAGCTCAGTGGTAGAGCGCTTGCCTAGCAAGCGCAAGG | 25201 | 0.11626168377790821 | No Hit |

## Adapter Content

Produced by FastQC (version 0.11.8)
