# Supplementary material for: Metabolic and Transcriptomic Changes in the Mouse Brain in Response to Short-Term High-Fat Metabolic Stress
Source: Metabolites. 2023 Mar 9;13(3):407. doi: 10.3390/metabo13030407 (PMC10051449; doi:10.3390/metabo13030407)
Supplement: Supplementary file 1 [file metabolites-13-00407-s001.zip › 230207_Metabolites_FastQC/NCD_1_fastqc.html]

NCD\_1.fastq.gz FastQC Report 

FastQC Report

월 22 8월 2022  
NCD\_1.fastq.gz

## Summary

- Basic Statistics
- Per base sequence quality
- Per tile sequence quality
- Per sequence quality scores
- Per base sequence content
- Per sequence GC content
- Per base N content
- Sequence Length Distribution
- Sequence Duplication Levels
- Overrepresented sequences
- Adapter Content

## Basic Statistics

| Measure | Value |
| --- | --- |
| Filename | NCD\_1.fastq.gz |
| File type | Conventional base calls |
| Encoding | Sanger / Illumina 1.9 |
| Total Sequences | 19151128 |
| Sequences flagged as poor quality | 0 |
| Sequence length | 76 |
| %GC | 45 |

## Per base sequence quality

## Per tile sequence quality

## Per sequence quality scores

## Per base sequence content

## Per sequence GC content

## Per base N content

## Sequence Length Distribution

## Sequence Duplication Levels

## Overrepresented sequences

| Sequence | Count | Percentage | Possible Source |
| --- | --- | --- | --- |
| GGGTTGGGGATTTAGCTCAGTGGTAGAGCGCTTGCCTAGCAAGCGCAAGG | 329468 | 1.720358195089083 | No Hit |
| GGTTGGGGATTTAGCTCAGTGGTAGAGCGCTTGCCTAGCAAGCGCAAGGC | 272636 | 1.4236028290344047 | No Hit |
| GGGGTTGGGGATTTAGCTCAGTGGTAGAGCGCTTGCCTAGCAAGCGCAAG | 257044 | 1.3421872591525679 | No Hit |
| TTGGGGATTTAGCTCAGTGGTAGAGCGCTTGCCTAGCAAGCGCAAGGCCC | 218266 | 1.1397031026057578 | No Hit |
| GTTGGGGATTTAGCTCAGTGGTAGAGCGCTTGCCTAGCAAGCGCAAGGCC | 171933 | 0.8977695726330063 | No Hit |
| GGGGATTTAGCTCAGTGGTAGAGCGCTTGCCTAGCAAGCGCAAGGCCCTG | 120716 | 0.6303336283899309 | No Hit |
| TGGGGATTTAGCTCAGTGGTAGAGCGCTTGCCTAGCAAGCGCAAGGCCCT | 94169 | 0.4917151616343434 | No Hit |
| GGGATTTAGCTCAGTGGTAGAGCGCTTGCCTAGCAAGCGCAAGGCCCTGG | 65360 | 0.3412853801614192 | No Hit |
| TGGGGTTGGGGATTTAGCTCAGTGGTAGAGCGCTTGCCTAGCAAGCGCAA | 58104 | 0.30339727247397646 | No Hit |
| AGGGTTGGGGATTTAGCTCAGTGGTAGAGCGCTTGCCTAGCAAGCGCAAG | 42910 | 0.22405990916044213 | No Hit |
| GGATTTAGCTCAGTGGTAGAGCGCTTGCCTAGCAAGCGCAAGGCCCTGGG | 38939 | 0.20332483809830942 | No Hit |
| AGGGGTTGGGGATTTAGCTCAGTGGTAGAGCGCTTGCCTAGCAAGCGCAA | 38322 | 0.2001030957549863 | No Hit |
| TGGGTTGGGGATTTAGCTCAGTGGTAGAGCGCTTGCCTAGCAAGCGCAAG | 37869 | 0.1977376998367929 | No Hit |
| GGGGTGGGGATTTAGCTCAGTGGTAGAGCGCTTGCCTAGCAAGCGCAAGG | 27807 | 0.14519771368036388 | No Hit |
| GGGTGGGGATTTAGCTCAGTGGTAGAGCGCTTGCCTAGCAAGCGCAAGGC | 26711 | 0.13947481318071708 | No Hit |
| AGGTTGGGGATTTAGCTCAGTGGTAGAGCGCTTGCCTAGCAAGCGCAAGG | 25503 | 0.13316709073220126 | No Hit |
| GCTCAGTGGTAGAGCGCTTGCCTAGCAAGCGCAAGGCCCTGGGTTCGGTC | 22960 | 0.11988849951814848 | No Hit |
| TGGTTGGGGATTTAGCTCAGTGGTAGAGCGCTTGCCTAGCAAGCGCAAGG | 22517 | 0.1175753198453898 | No Hit |
| GATTTAGCTCAGTGGTAGAGCGCTTGCCTAGCAAGCGCAAGGCCCTGGGT | 21846 | 0.11407160977671917 | No Hit |

## Adapter Content

Produced by FastQC (version 0.11.8)
