# Supplementary material for: Metabolic and Transcriptomic Changes in the Mouse Brain in Response to Short-Term High-Fat Metabolic Stress
Source: Metabolites. 2023 Mar 9;13(3):407. doi: 10.3390/metabo13030407 (PMC10051449; doi:10.3390/metabo13030407)
Supplement: Supplementary file 1 [file metabolites-13-00407-s001.zip › 230207_Metabolites_FastQC/NCD_2_fastqc.html]

NCD\_2.fastq.gz FastQC Report 

FastQC Report

월 22 8월 2022  
NCD\_2.fastq.gz

## Summary

- Basic Statistics
- Per base sequence quality
- Per tile sequence quality
- Per sequence quality scores
- Per base sequence content
- Per sequence GC content
- Per base N content
- Sequence Length Distribution
- Sequence Duplication Levels
- Overrepresented sequences
- Adapter Content

## Basic Statistics

| Measure | Value |
| --- | --- |
| Filename | NCD\_2.fastq.gz |
| File type | Conventional base calls |
| Encoding | Sanger / Illumina 1.9 |
| Total Sequences | 21508147 |
| Sequences flagged as poor quality | 0 |
| Sequence length | 76 |
| %GC | 44 |

## Per base sequence quality

## Per tile sequence quality

## Per sequence quality scores

## Per base sequence content

## Per sequence GC content

## Per base N content

## Sequence Length Distribution

## Sequence Duplication Levels

## Overrepresented sequences

| Sequence | Count | Percentage | Possible Source |
| --- | --- | --- | --- |
| GGGTTGGGGATTTAGCTCAGTGGTAGAGCGCTTGCCTAGCAAGCGCAAGG | 342459 | 1.5922292143530543 | No Hit |
| GGTTGGGGATTTAGCTCAGTGGTAGAGCGCTTGCCTAGCAAGCGCAAGGC | 286856 | 1.3337085709894023 | No Hit |
| GGGGTTGGGGATTTAGCTCAGTGGTAGAGCGCTTGCCTAGCAAGCGCAAG | 255569 | 1.1882427621496172 | No Hit |
| TTGGGGATTTAGCTCAGTGGTAGAGCGCTTGCCTAGCAAGCGCAAGGCCC | 229022 | 1.0648151140123787 | No Hit |
| GTTGGGGATTTAGCTCAGTGGTAGAGCGCTTGCCTAGCAAGCGCAAGGCC | 180217 | 0.8379010985930122 | No Hit |
| GGGGATTTAGCTCAGTGGTAGAGCGCTTGCCTAGCAAGCGCAAGGCCCTG | 120475 | 0.5601365845230646 | No Hit |
| TGGGGATTTAGCTCAGTGGTAGAGCGCTTGCCTAGCAAGCGCAAGGCCCT | 93414 | 0.43431914427588764 | No Hit |
| GGGATTTAGCTCAGTGGTAGAGCGCTTGCCTAGCAAGCGCAAGGCCCTGG | 65212 | 0.30319673749672627 | No Hit |
| TGGGGTTGGGGATTTAGCTCAGTGGTAGAGCGCTTGCCTAGCAAGCGCAA | 56296 | 0.26174267825117614 | No Hit |
| AGGGTTGGGGATTTAGCTCAGTGGTAGAGCGCTTGCCTAGCAAGCGCAAG | 46088 | 0.21428159292383486 | No Hit |
| TGGGTTGGGGATTTAGCTCAGTGGTAGAGCGCTTGCCTAGCAAGCGCAAG | 40049 | 0.18620386033255212 | No Hit |
| GGATTTAGCTCAGTGGTAGAGCGCTTGCCTAGCAAGCGCAAGGCCCTGGG | 39390 | 0.18313990507875924 | No Hit |
| AGGGGTTGGGGATTTAGCTCAGTGGTAGAGCGCTTGCCTAGCAAGCGCAA | 39335 | 0.18288418802419382 | No Hit |
| AGGTTGGGGATTTAGCTCAGTGGTAGAGCGCTTGCCTAGCAAGCGCAAGG | 27851 | 0.12949046703093484 | No Hit |
| GGGGTGGGGATTTAGCTCAGTGGTAGAGCGCTTGCCTAGCAAGCGCAAGG | 27843 | 0.12945327182299804 | No Hit |
| GGGTGGGGATTTAGCTCAGTGGTAGAGCGCTTGCCTAGCAAGCGCAAGGC | 27080 | 0.1259057788660269 | No Hit |
| TGGTTGGGGATTTAGCTCAGTGGTAGAGCGCTTGCCTAGCAAGCGCAAGG | 24450 | 0.1136778542568079 | No Hit |
| GCTCAGTGGTAGAGCGCTTGCCTAGCAAGCGCAAGGCCCTGGGTTCGGTC | 22678 | 0.10543911569880937 | No Hit |
| CCAACCCTCACACACACGAGAACTAACACTAATAGCCCTTCACATAATTC | 22231 | 0.10336083345534137 | No Hit |

## Adapter Content

Produced by FastQC (version 0.11.8)
