# Supplementary material for: Metabolic and Transcriptomic Changes in the Mouse Brain in Response to Short-Term High-Fat Metabolic Stress
Source: Metabolites. 2023 Mar 9;13(3):407. doi: 10.3390/metabo13030407 (PMC10051449; doi:10.3390/metabo13030407)
Supplement: Supplementary file 1 [file metabolites-13-00407-s001.zip › 230207_Metabolites_FastQC/NCD_3_fastqc.html]

NCD\_3.fastq.gz FastQC Report 

FastQC Report

월 22 8월 2022  
NCD\_3.fastq.gz

## Summary

- Basic Statistics
- Per base sequence quality
- Per tile sequence quality
- Per sequence quality scores
- Per base sequence content
- Per sequence GC content
- Per base N content
- Sequence Length Distribution
- Sequence Duplication Levels
- Overrepresented sequences
- Adapter Content

## Basic Statistics

| Measure | Value |
| --- | --- |
| Filename | NCD\_3.fastq.gz |
| File type | Conventional base calls |
| Encoding | Sanger / Illumina 1.9 |
| Total Sequences | 22740197 |
| Sequences flagged as poor quality | 0 |
| Sequence length | 76 |
| %GC | 47 |

## Per base sequence quality

## Per tile sequence quality

## Per sequence quality scores

## Per base sequence content

## Per sequence GC content

## Per base N content

## Sequence Length Distribution

## Sequence Duplication Levels

## Overrepresented sequences

| Sequence | Count | Percentage | Possible Source |
| --- | --- | --- | --- |
| GGGTTGGGGATTTAGCTCAGTGGTAGAGCGCTTGCCTAGCAAGCGCAAGG | 529093 | 2.3266860880756663 | No Hit |
| GGTTGGGGATTTAGCTCAGTGGTAGAGCGCTTGCCTAGCAAGCGCAAGGC | 440765 | 1.9382637714176354 | No Hit |
| GGGGTTGGGGATTTAGCTCAGTGGTAGAGCGCTTGCCTAGCAAGCGCAAG | 435197 | 1.9137784954105719 | No Hit |
| TTGGGGATTTAGCTCAGTGGTAGAGCGCTTGCCTAGCAAGCGCAAGGCCC | 331015 | 1.4556382251217965 | No Hit |
| GTTGGGGATTTAGCTCAGTGGTAGAGCGCTTGCCTAGCAAGCGCAAGGCC | 280570 | 1.2338063737970255 | No Hit |
| GGGGATTTAGCTCAGTGGTAGAGCGCTTGCCTAGCAAGCGCAAGGCCCTG | 171657 | 0.7548615344009553 | No Hit |
| TGGGGATTTAGCTCAGTGGTAGAGCGCTTGCCTAGCAAGCGCAAGGCCCT | 157274 | 0.6916123022153238 | No Hit |
| TGGGGTTGGGGATTTAGCTCAGTGGTAGAGCGCTTGCCTAGCAAGCGCAA | 125147 | 0.5503338427543086 | No Hit |
| GGGATTTAGCTCAGTGGTAGAGCGCTTGCCTAGCAAGCGCAAGGCCCTGG | 88284 | 0.3882288266895841 | No Hit |
| TGGGTTGGGGATTTAGCTCAGTGGTAGAGCGCTTGCCTAGCAAGCGCAAG | 70595 | 0.3104414618747586 | No Hit |
| AGGGTTGGGGATTTAGCTCAGTGGTAGAGCGCTTGCCTAGCAAGCGCAAG | 69570 | 0.3059340251098089 | No Hit |
| AGGGGTTGGGGATTTAGCTCAGTGGTAGAGCGCTTGCCTAGCAAGCGCAA | 62748 | 0.27593428500201644 | No Hit |
| GGGGTGGGGATTTAGCTCAGTGGTAGAGCGCTTGCCTAGCAAGCGCAAGG | 52894 | 0.23260132706853862 | No Hit |
| GGATTTAGCTCAGTGGTAGAGCGCTTGCCTAGCAAGCGCAAGGCCCTGGG | 50309 | 0.22123379142229946 | No Hit |
| GGGTGGGGATTTAGCTCAGTGGTAGAGCGCTTGCCTAGCAAGCGCAAGGC | 47235 | 0.20771587862673307 | No Hit |
| AGGTTGGGGATTTAGCTCAGTGGTAGAGCGCTTGCCTAGCAAGCGCAAGG | 42641 | 0.18751376692119245 | No Hit |
| TGGTTGGGGATTTAGCTCAGTGGTAGAGCGCTTGCCTAGCAAGCGCAAGG | 41357 | 0.18186737784197735 | No Hit |
| CGGGGTTGGGGATTTAGCTCAGTGGTAGAGCGCTTGCCTAGCAAGCGCAA | 36347 | 0.15983590643475956 | No Hit |
| GCTCAGTGGTAGAGCGCTTGCCTAGCAAGCGCAAGGCCCTGGGTTCGGTC | 32221 | 0.14169182439360575 | No Hit |
| GGTGGGGATTTAGCTCAGTGGTAGAGCGCTTGCCTAGCAAGCGCAAGGCC | 30463 | 0.13396102065430657 | No Hit |
| GTGGTTGGGGATTTAGCTCAGTGGTAGAGCGCTTGCCTAGCAAGCGCAAG | 28671 | 0.12608070193938953 | No Hit |
| GATTTAGCTCAGTGGTAGAGCGCTTGCCTAGCAAGCGCAAGGCCCTGGGT | 28472 | 0.12520559958209684 | No Hit |
| GGTGTTGGGGATTTAGCTCAGTGGTAGAGCGCTTGCCTAGCAAGCGCAAG | 27125 | 0.11928216804806045 | No Hit |
| TGTTGGGGATTTAGCTCAGTGGTAGAGCGCTTGCCTAGCAAGCGCAAGGC | 26248 | 0.1154255611769766 | No Hit |
| TTGGGGTTGGGGATTTAGCTCAGTGGTAGAGCGCTTGCCTAGCAAGCGCA | 25809 | 0.11349505899179324 | No Hit |
| GTGGGTTGGGGATTTAGCTCAGTGGTAGAGCGCTTGCCTAGCAAGCGCAA | 25737 | 0.11317843904342605 | No Hit |
| TAGCTCAGTGGTAGAGCGCTTGCCTAGCAAGCGCAAGGCCCTGGGTTCGG | 23354 | 0.10269919825232825 | No Hit |
| GGGTGTTGGGGATTTAGCTCAGTGGTAGAGCGCTTGCCTAGCAAGCGCAA | 22988 | 0.10108971351479497 | No Hit |

## Adapter Content

Produced by FastQC (version 0.11.8)
