# Supplementary material for: Metabolic and Transcriptomic Changes in the Mouse Brain in Response to Short-Term High-Fat Metabolic Stress
Source: Metabolites. 2023 Mar 9;13(3):407. doi: 10.3390/metabo13030407 (PMC10051449; doi:10.3390/metabo13030407)
Supplement: Supplementary file 1 [file metabolites-13-00407-s001.zip › 230207_Metabolites_SUPPLEMENTARY FIGURE_Kim_Hong.pptx]

## Slide 1
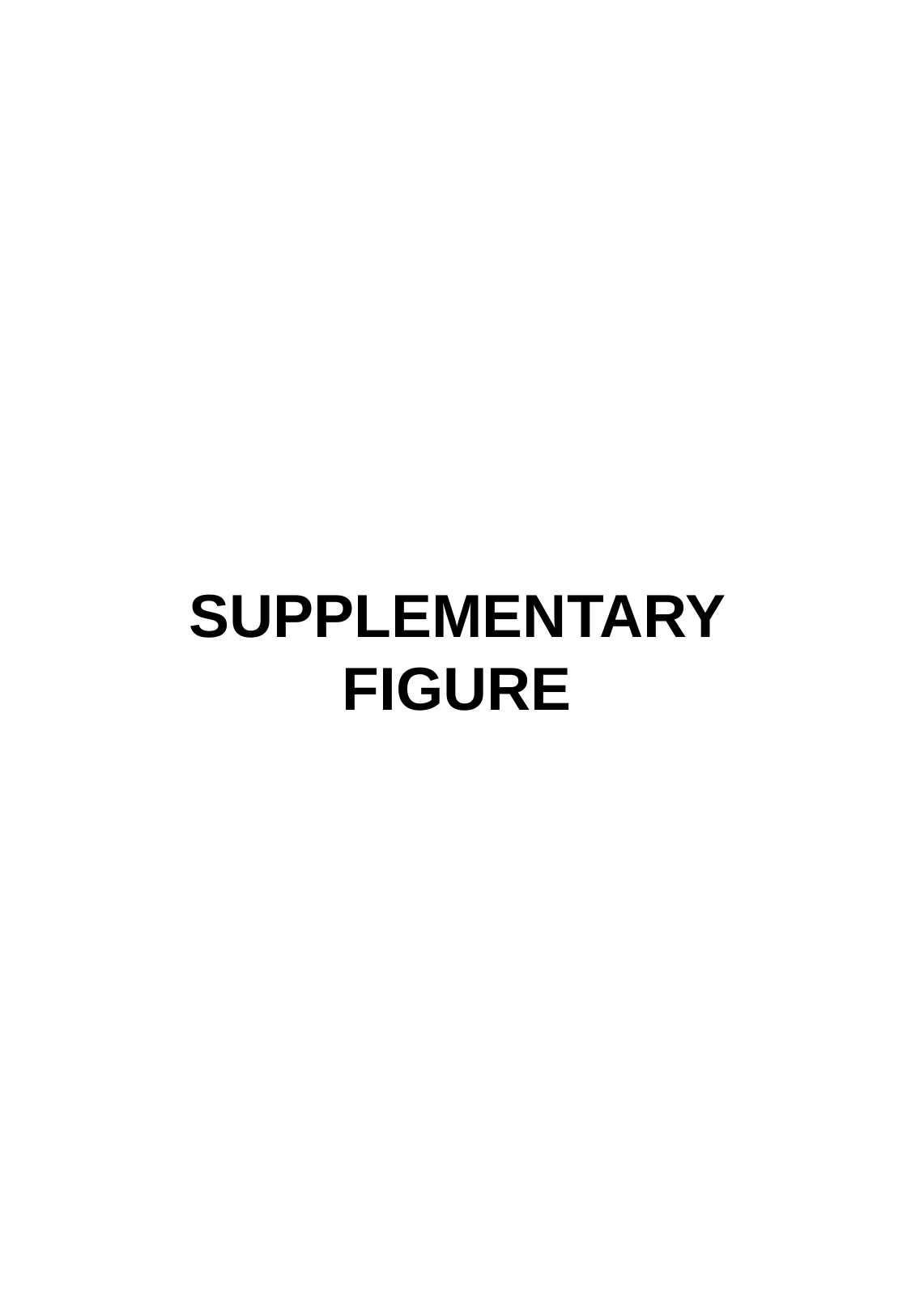

SUPPLEMENTARY FIGURE

## Slide 2
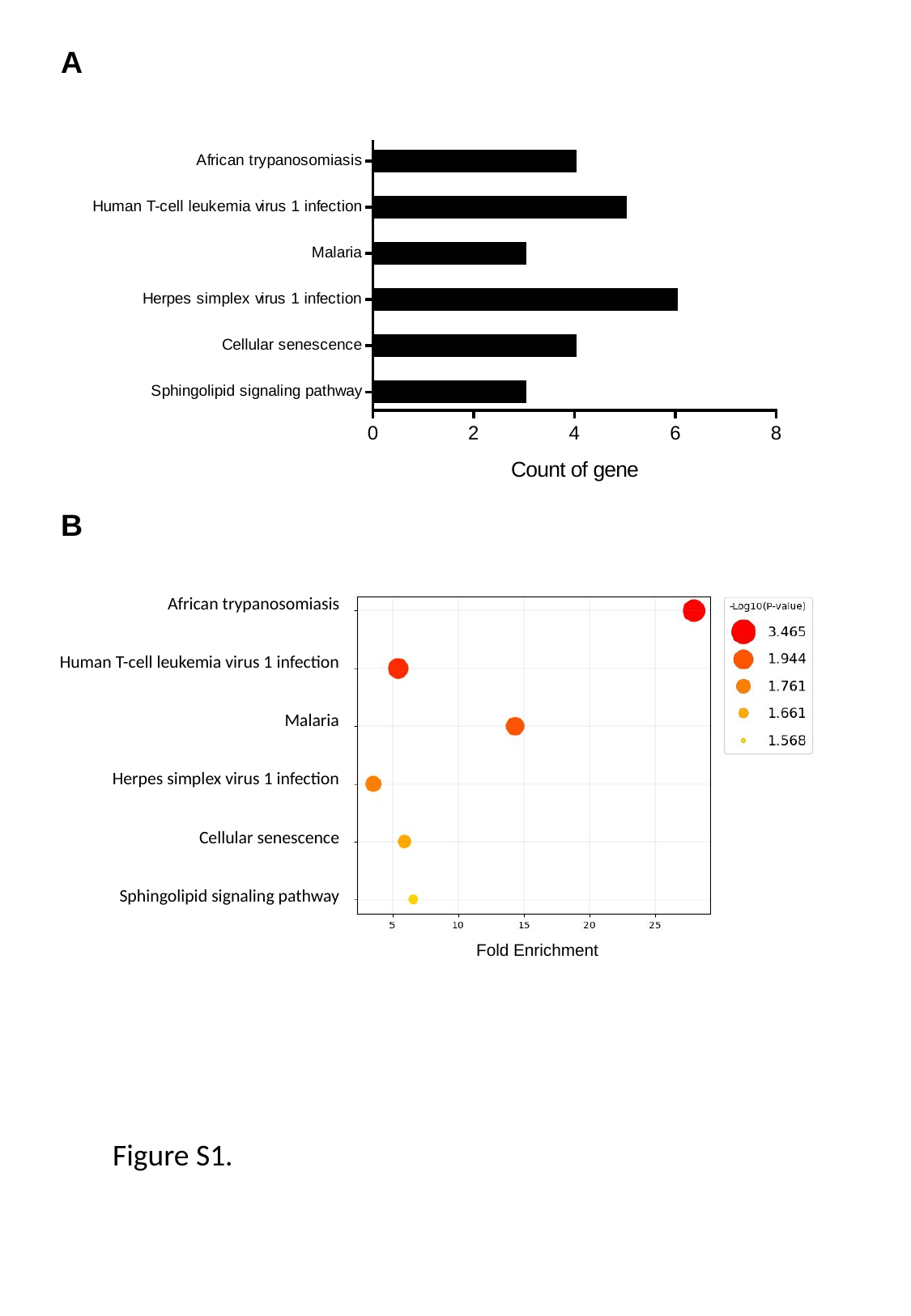

A
B
| African trypanosomiasis |
| --- |
| Human T-cell leukemia virus 1 infection |
| Malaria |
| Herpes simplex virus 1 infection |
| Cellular senescence |
| Sphingolipid signaling pathway |
Fold Enrichment
Figure S1.

## Slide 3
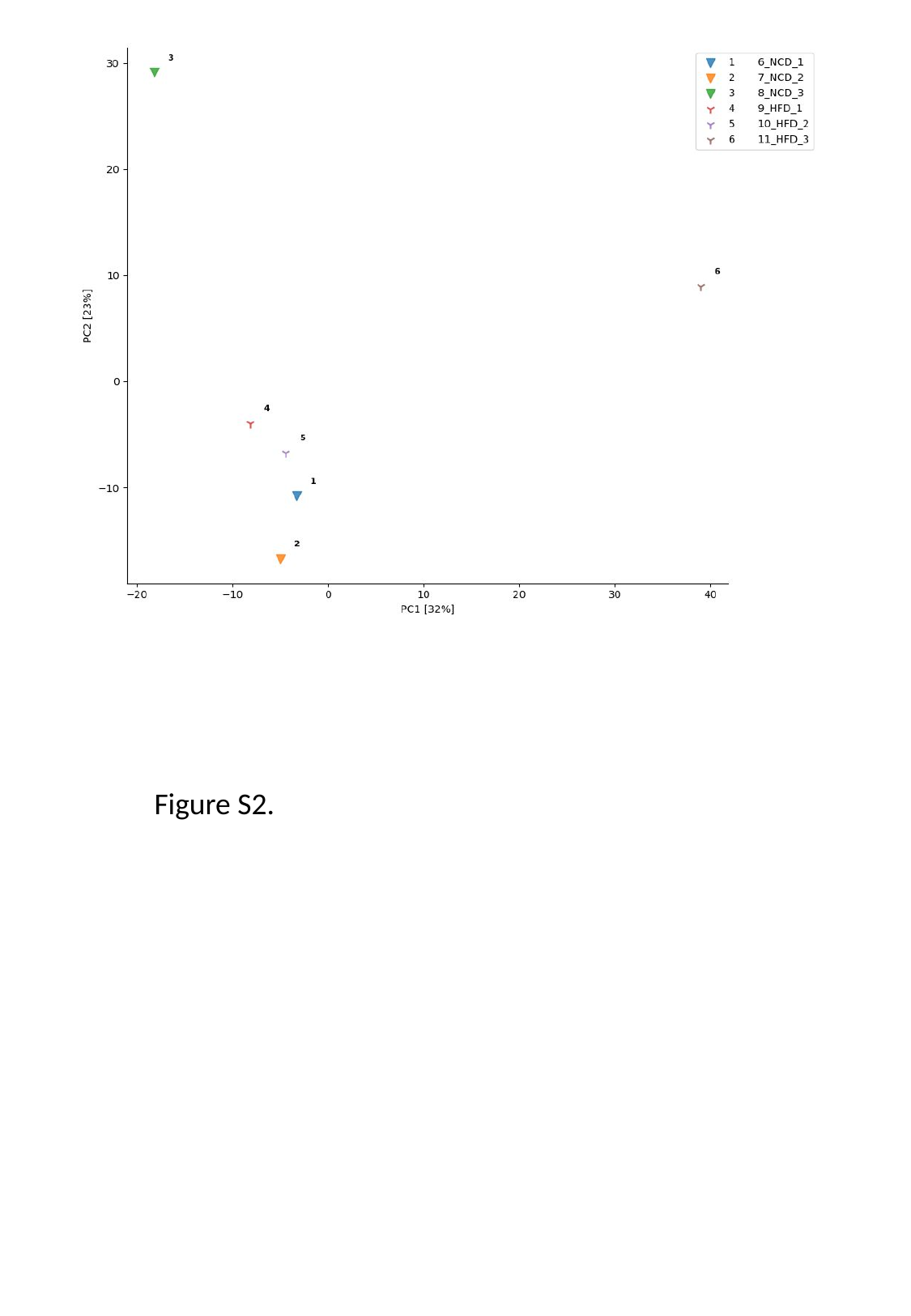

Figure S2.
